# Clinical and Genetic Characteristics of Mitochondrial DNA Depletion Syndrome Associated with *SUCLG1* Variants in China

**DOI:** 10.33549/physiolres.935640

**Published:** 2026-04-01

**Authors:** Gai-Xiu ZHANG, Ke-Jia JI, Zi-Wei WANG, Ni-Na WANG

**Affiliations:** 1 Department of Endocrine Genetics and Metabolism, Shanxi Children’s Hospital, Taiyuan, Shanxi Province, China; 2 Department of Pediatrics, Beijing Haidian Hospital, Beijing, China; 3 Shanxi Medical University, Taiyuan, Shanxi Province, China

**Keywords:** China, Clinical features, Methylmalonic aciduria, Mitochondrial DNA depletion syndrome, *SUCLG1* gene

## Abstract

This study aimed to summarize the genetic variants and clinical characteristics of mitochondrial DNA depletion syndrome (MDS) associated with *SUCLG1* mutations in children from China. A systematic review of cases reported in a Chinese literature database was conducted. Clinical data and genetic findings of children with MDS caused by *SUCLG1* mutations were analyzed. A total of 13 cases from 9 articles were identified. The primary clinical features included hypotonia, psychomotor retardation, feeding difficulties, growth retardation, hearing impairment, and liver function impairment. Urine organic acid analysis demonstrated a mild increase in methylmalonic acid, while plasma concentrations of propionylcarnitine and/or butyrylcarnitine were elevated. Additionally, increased lactate and pyruvic acid levels were observed in both plasma and cerebrospinal fluid. Brain magnetic resonance imaging identified basal ganglion lesions and/or cerebral atrophy. A total of 14 *SUCLG1* variants were identified: c.550G>A, c.751C>T, c.809A>C, c.961C>G, c.826-2A>G, c.713T>C, c.916G>T, c.619T>C, c.980dupT, c.40A>G, c.142C>T, c.601A>G, c.871G>C, and c.721_c.722delGA. Among these, the c.826-2A>G variation was the most frequently detected, present in 4 children, followed by c.550G>A. No significant correlation was found between genotype and phenotype. All 13 children were treated with vitamin B complex and coenzyme Q10. Among them, 2 died, while the remaining children exhibited clinical improvement. MDS associated with *SUCLG1* mutations presents with nonspecific clinical manifestations and can affect multiple organ systems. Genetic testing is necessary for diagnosis, and no definitive treatment is currently available.

## Introduction

Mitochondrial DNA depletion syndrome (MDS) is a rare mitochondrial disorder characterized by a severe reduction in mitochondrial DNA (mtDNA) resulting from nuclear gene mutations, leading to impaired energy production in affected tissues and organs [1,2]. The condition was first described by Moraes *et al.* in 1991 [3]. MDS follows an autosomal recessive inheritance pattern, with complex and diverse genotypes and clinical phenotypes. It is classified into four clinical subtypes: myopathy, encephalomyopathy, hepatocerebral type, and neurogastrointestinal myopathy, each associated with distinct genetic mutations.

The encephalomyopathy subtype primarily manifests in infancy, presenting with hypotonia and neurological dysfunction. The causative genes include *SUCLA2*, *SUCLG1*, and *RRM2B* [4]. Encephalomyopathy caused by *SUCLG1* mutations, also referred to as mitochondrial DNA depletion syndrome 9 (MTDPS9) (OMIM #245400, encephalomyopathy with methylmalonic aciduria), currently lacks an effective treatment [5]. The prognosis is poor, with mortality frequently stemming from complications attributable to concurrent infections.

To date, over 40 cases of *SUCLG1* variants have been reported worldwide, while only 13 cases of MTDPS9 have been documented in China [1,4–12]. This article reviews nine clinical reports on MTDPS9 in China, summarizing the clinical characteristics of 13 affected children to enhance clinicians’ understanding of MTDPS9 associated with *SUCLG1* mutations.

## Methods

### Literature search

A comprehensive search was conducted using the terms ‘Mitochondrial DNA depletion syndrome’ and ‘*SUCLG1* gene variation’ in Chinese literature databases, including CNKI, VIP, Wanfang Database, and China Biomedical Literature Database. Full-text and subject heading searches were performed to identify all original case reports of MTDPS9 associated with *SUCLG1* mutations. Each identified study was manually reviewed, and only cases confirmed through genetic testing were included. Studies on MDS caused by mutations in genes other than *SUCLG1* and review articles were excluded. Additionally, when multiple reports originated from the same institution, only the most comprehensive dataset was selected. Ultimately, nine articles comprising 13 cases were included in the analysis.

### Statistical analysis

A structured Excel database was created to record demographic and clinical data, including sex, age, gene mutation sites, clinical manifestations, laboratory findings, and imaging results of the included cases.

## Results

### Sex, age

Among the 13 children diagnosed with MTDPS9 caused by *SUCLG1* mutations, nine were male and four were female, resulting in a male-to-female ratio of 9:4. Disease onset occurred between the first day after birth and six months of age in all cases.

### Clinical manifestations and signs

The most commonly reported initial symptoms included motor development delay (7/13), feeding difficulties (6/13), hypotonia (8/13), and growth retardation (6/13). The primary clinical manifestations observed were psychomotor retardation (10/13), hypotonia (13/13), growth retardation (11/13), feeding difficulties (9/13), hearing impairment (9/13), liver dysfunction (9/13), epilepsy (3/13), and scoliosis or kyphotic deformity (2/13) (Table 1).

### Laboratory examinations

Serum lactate levels ranged from 2.10 to 13.7 mmol/L, and pyruvic acid levels ranged from 127–319 mmol/L with varying degrees of elevation across cases. Liver function tests indicated increased alanine aminotransferase (ALT) (41–116 IU/L) and aspartate aminotransferase (AST) (40–149 IU/L) in most cases. Homocysteine levels remained within the normal range in all cases. Urine organic acid analysis indicated a slight increase in methylmalonic acid (6.5–9.97 mmol/mol Cr) (1.80–30.7 times), with a few cases showing elevated 3-hydroxypropionic acid and methyl citrate concentrations. Amino acid and acylcarnitine analysis for inherited metabolic disorders demonstrated increased plasma propio-nylcarnitine (3.41–6.45 μmol/L) and succinyl-carnitine (0.06–4.49 μmol/L) in most of the children (Table 2).

### Imaging examinations

Magnetic resonance imaging (MRI) findings were available for 13 children, of whom 5 exhibited concurrent basal ganglia lesions and cerebral atrophy. Leigh-like changes were observed, characterized by bilateral atrophy of the lenticular nucleus, putamen, and caudate nucleus, accompanied by long T2 signal hyperintensity.

### Genetic testing

Diagnosis in all cases was confirmed through genetic testing. To date, all 13 reported cases in China were non-consanguineous. A total of 14 SUCLG1-related pathogenic variants were identified: c.550G>A, c.751C>T, c.809A>C, c.961C>G, c.826-2A>G, c.713T>C, c.916G>T, c.619T>C, c.980dupT, c.40A>G, c.142C>T, c.601A>G, c.871G>C, and c.721_c.722delGA. Among these, the c.826-2A>G variant was the most frequently detected (5/26), followed by c.550G>A (4/26). No significant correlation was found between genotype and phenotype.

### Treatment

Following the diagnosis of MTDPS9 caused by *SUCLG1* mutations, all 13 children were treated with levocarnitine, vitamin B complex, and coenzyme Q10. Among them, two patients (cases 7 and 9) died due to multi-organ failure. One case (case 7) succumbed at 8 months of disease progression, while the other (case 9) died 11 days after birth due to severe lactic acidosis, progressive dyspnea, and edema. The remaining children showed clinical improvement after treatment.

## Discussion

MDS represents a clinically heterogeneous group of multisystem disorders characterized by a severe reduction in mitochondrial DNA due to nuclear gene mutations. The condition affects the brain, heart, kidneys, and liver, with varying severity. Clinically, it is categorized into four types based on the primary organs involved and the associated pathogenic genes. The myopathic subtype, caused by *TK2* mutations, is characterized by hypotonia, muscle weakness, and exercise intolerance [13]. The encephalomyopathy subtype, associated with *SUCLA2*, *SUCLG1*, and *RRM2B* mutations, typically manifests in infancy with hypotonia and neurological dysfunction [5]. The hepatocerebral subtype, linked to mutations in *DGUOK*, *MPV17*, *POLG*, and *C10orf2*, presents either as severe hepatic encephalopathy with neonatal or infantile onset or as spinocerebellar ataxia beginning in infancy. The neurogastrointestinal myopathy subtype, caused by *TYMP* mutations, is characterized by ptosis, ophthalmoplegia, peripheral neuropathy, leukoencephalopathy, gastrointestinal motility disorders, and cachexia.

MTDPS9 is caused by mutations in the *SUCLG1* gene, which is located on chromosome 2p11.3 and comprises nine exons encoding a 346-amino-acid SUCLG1 protein [14]. In China, *SUCLG1*-related MDS was first reported by Liu *et al.* in 2016 [6]. Mutations in *SUCLG1* cause dysfunction of succinyl-CoA ligase (SUCL), a rate-limiting enzyme in the tricarboxylic acid cycle responsible for catalyzing the conversion of succinyl-CoA and ADP/GDP to succinic acid and ATP/GTP [13]. SUCL dysfunction disrupts this process, leading to succinyl-CoA accumulation and impairing its production from methylmalonyl-CoA, thereby increasing methylmalonic acid levels [14]. Additionally, SUCL interacts with mitochondrial nucleoside diphosphate kinases, contributing to nucleotide production. Its dysfunction results in the deficiency of this complex, affecting mtDNA synthesis and ultimately leading to mtDNA depletion [14].

The SUCL protein consists of α and β subunits, with the α subunit encoded by the *SUCLG1* gene and the β subunit encoded by the *SUCLA2* and *SUCLG2* genes. All three genes contribute to the tricarboxylic acid cycle. *SUCLG1* is widely expressed, with the highest levels in the heart, brain, kidney, and liver. *SUCLG2* is predominantly expressed in biosynthetic tissues, particularly the liver and kidney, whereas *SUCLA2* is primarily found in the heart, skeletal muscle, and brain [15]. Mutations in *SUCLA2* and *SUCLG1* lead to the encephalomyopathy subtype of MDS. Due to SUCL protein deficiency, patients with this type of MDS typically exhibit severe clinical manifestations, including hypotonia, muscle atrophy, and psychomotor retardation. Additional common features include progressive scoliosis or kyphosis, abnormal movements (dystonia, athetoid movements, or chorea), growth retardation, feeding difficulties, gastroesophageal reflux, sensorineural hearing impairment, and liver dysfunction. Some patients may also present with hypertrophic cardiomyopathy, recurrent respiratory tract infections, hyperhidrosis, strabismus, ptosis, sleep disturbances, and epilepsy [2].

Urine organic acid analysis typically demonstrates a mild increase in methylmalonic acid, along with elevated plasma propionylcarnitine and/or butyrylcarnitine levels. Lactate concentrations are elevated in both plasma and cerebrospinal fluid, and electromyography often indicate motor neuron involvement. Brain MRI findings include bilateral basal ganglia hyperintensity (80 %), cerebral atrophy (30 %), and leukoencephalopathy (20 %) [10]. Histopathological analysis of skeletal muscle shows increased fiber variability, a higher mitochondrial count, and extensive intracellular fat accumulation. Mitochondrial respiratory electron transport chain (ETC) enzyme activity in muscle usually demonstrates a combined deficiency of complexes I, III, and IV, while complex II activity remains normal. Quantitative mitochondrial DNA analysis indicates reduced mtDNA levels in muscle. However, some reports suggest that children with *SUCLG1* mutations may exhibit severe symptoms despite normal ETC activity and/or the absence of mtDNA deletion, implying the involvement of additional, yet unidentified, pathogenic mechanisms [16].

Compared with affected children in other countries, the majority of cases reported in China involve male children. Hypotonia, growth retardation, and psychomotor delay are the most prevalent symptoms, followed by feeding difficulties, hearing impairment, and liver dysfunction. In contrast, recurrent respiratory tract infections, hyperhidrosis, epilepsy, scoliosis, and kyphosis are less frequently observed in Chinese children. Brain MRI findings indicate a similar incidence of basal ganglia lesions and cerebral atrophy (both 50 %). No significant differences have been noted between Chinese children and those from other regions regarding biochemical test results or urine metabolic screening.

Mutations in both *SUCLG1* and *SUCLA2* are associated with encephalomyopathy subtype of MDS, often accompanied by mild methylmalonic aciduria. However, the clinical presentation varies depending on the specific genetic mutation. *SUCLG1* mutations are less common but result in a more severe phenotype and shorter survival compared to *SUCLA2*-related mitochondrial diseases [7, 17]. Ostergaard *et al.* reported that *SUCLA2*-associated MDS generally presents with a milder disease course, with affected children being asymptomatic at birth, experiencing symptom onset in infancy or later, and having a life expectancy of up to 20 years due to residual G-SUCL function. In contrast, *SUCLG1* mutations cause complete SUCL deficiency, leading to a more severe condition, with most affected children succumbing in the neonatal period and an average survival of approximately 20 months [5,18].

However, some reports indicate that children with *SUCLG1* mutations may survive into their twenties, suggesting that life expectancy may also be influenced by individual variability [15]. Additionally, Carrozzo *et al.* observed that hypertrophic cardiomyopathy and hepatic involvement occurred exclusively in children with *SUCLG1* mutations, whereas epilepsy was more prevalent among those with *SUCLA2* mutations [17]. Ostergaard *et al.* further identified a patient with a novel homozygous *SUCLG1* missense variant (c.215G>C) who exhibited mild symptoms resembling those seen in *SUCLA2*-related MDS, possibly due to a higher residual level of SUCLG1 protein [19, 20]. Given the overlapping clinical features of SUCLG1- and SUCLA2-related MDS, genetic sequencing remains crucial for differential diagnosis when clinical manifestations alone are insufficient.

Liver dysfunction is a common clinical feature in children with MTDPS9. Studies have reported that approximately 40 % of patients with *SUCLG1* mutations exhibit hepatic involvement, characterized by hepatomegaly, steatosis, elevated liver enzymes, liver failure, or unspecified liver abnormalities [14]. This is attributed to the high expression of the *SUCLG1* gene in liver tissue, which relies heavily on ATP biosynthesis and detoxification. Consequently, mitochondrial dysfunction frequently results in hepatic impairment [21].

Timely administration of hepatoprotective therapy is essential for managing liver dysfunction. In cases of liver failure, liver transplantation serves as a viable treatment approach to enhance both quality of life and life expectancy. Hegarty *et al.* described a 17-year-old child with MDS caused by *SUCLG1* and *POLG* mutations who developed acute liver failure at 17 months of age, underwent liver transplantation, and currently exhibited mild left ventricular hypertrophy without other complications [22].

Additionally, children with MTDPS9 frequently experience growth retardation, and liver transplantation has demonstrated to promote height gain. Children with hereditary metabolic disorders often exhibit growth and developmental delays prior to transplantation, but these parameters improve post-transplant. Notably, significant increases in height and weight Z-scores are observed within a year following the procedure. However, in children with severe neurological symptoms, such as dystonia, psychomotor retardation, or nystagmus, liver transplantation is not recommended.

The prognosis of SUCLG1-related MTDPS9 is poor, with most affected children succumbing to respiratory failure due to co-infection during childhood or even the neonatal period [2]. Additionally infertility has been reported in affected children who survive into adulthood [14]. Currently, no effective curative treatment exists. Supportive therapies, including levocarnitine, vitamin B complex, and coenzyme Q10, have been utilized, though their efficacy remains uncertain. Hydrolyzed protein and medium-chain fatty acid-enriched formulations aid in nutritional development [6]. Liver transplantation is a potential treatment for patients with liver failure. Growth hormone therapy facilitates height growth and normal development in affected children [23]. For patients with comorbid epilepsy, valproic acid should be avoided in combination with antiepileptic drugs, as it may exacerbate liver injury [9].

In summary, encephalomyopathic MDS is a rare disorder with nonspecific clinical manifestations, involving multiple systems and presenting significant diagnostic challenges. Genetic testing is essential for confirmation, and a high index of suspicion is warranted in children presenting with growth retardation, hypotonia, hearing impairment, hyperlactic acidemia, and mild methylmalonic aciduria [5]. Early recognition and diagnosis are crucial for prognosis assessment and optimizing quality-of-life.

## Figures and Tables

**Table 1:** General characteristics, clinical manifestations, and imaging findings of 13 children with MTDPS9

Case			1	2	3	4	5	6	7	8	9	10	11	12	13
*Author*			Liu et al. [7]			Lu et al. [5]	Hu et al.[1]		Dai LF et al. [4]		Zhang et al. [9]	Shen et al. [8]	Chen et al. [10]	Yao [11]	Ji [12]
*Sex*			Male	Male	Female	Male	Male	Male	Female	Male	Female	Male	Male	Male	Female
*Age of disease onset*			6-month-old	4-month-old	Newborn	4-month-old	At birth	At birth	At birth	At birth	2d after birth	3-month-old	1d after birth	4-month-old	4-month-old
*Gene mutation site*			C.826-2A>GC.809A>C	C.826-2A>GC.550G>A	C.961C>GC.751C>T	C.961C>GC.713T>C	C.826-2A>G	C.826-2A>GC.550G>A	C.916G>T	C.619T>CC.980dupT	C.40A>G	C.550G>AC.142C>T	C.601A>GC.871G>C	C.961C>GC.713T>C	C.550G>Ac.721_c.722delGA
*Initial symptoms*	Motor retardation		+	+	+	−	+	−	−	−	−	+	−	+	+
	Feeding difficulties		−	+	−	−	+	+	+	+	+	−	−	−	−
	Hypotonia		+	+	+	+	−	−	−	−	+	+	+	−	+
	Growth retardation		−	+	−	−	+	+	+	+	−	−	−	−	+
*Main symptoms*	Psychomotor retardation		+	+	+	+	+	+	−	−	−	+	+	+	+
	Hypotonia		+	+	+	+	+	+	+	+	+	+	+	+	+
	Growth retardation		+	+	+	+	+	+	+	+	−	−	+	+	+
	Feeding difficulties		+	+	+	+	+	+	+	+	+	−	−	−	−
	Hearing loss		−	−	−	+	+	+	+	+	−	+	+	+	+
	Liver function impairment		+	+	+	+	−	−	+	−	+	+	−	+	+
	Epilepsy		−	+	−	−	−	+	+	−	−	−	−	−	−
	Scoliosis kyphotic deformity		−	−	−	+	+	−	−	−	−	−	−	−	−
*Imaging examinations*	Brain MRI	Basal ganglion lesions	−	+	+	−	−	+	−	+	−	+	−	+	−
		Brain atrophy	−	+	−	+	−	+	−	+	+	+	−	+	−

Note: MRI: magnetic resonance imaging; + for yes; - for no

**Table 2:** Laboratory findings of 12 children with MTDPS9

Case	Blood										Urine		

	ALT (0~40IU/L)	AST (0~45IU/L)	Lactic acid 0.5-2 mmol/L	Pyruvic acid (30-100 mmol/L)	Homo-cysteine <15 μmol/L	C0 20~50 μmol/L	C2 6~30 μmol/L	C3 0.5~4 μmol/L	C4-DC 0.01~1 μmol/L	Creatine kinase 40-200 U/L	MMA 0.2~3.6 mmol/mol Cr	3-Hydroxy-propionic acid 0~1.1 mmol/mol Cr	Methyl citrate 0~0.7 mmol/mol Cr
*1*	100↑	41	4.0↑	255↑	N	66.57↑	No	5.07↑	4.27↑	No	9.97↑	3.04↑	No
*2*	41↑	40	4.1↑	140↑	N	20.8	No	5.09↑	1.98↑	No	9.21↑	4.54↑	No
*3*	51↑	69↑	4.8↑	319↑	N	63.8	No	5.33↑	4.49↑	No	8.03↑	3.15↑	No
*4*	49↑	65↑	2.10↑	127↑	N	No	47.47↑	No	4.44↑	206↑	Mild ↑	Mild ↑	Mild ↑
*5*	No	No	10.40↑	No	No	No	No	↑	No	No	Moderate ↑	No	No
*6*	N	No	3.4~4.1↑	No	No	No	No	No	No	N	Mild ↑ (13.8–30.7 times)	No	No
*7*	116↑	No	2.9~10.6↑	No	No	No	No	No	No	N	Mild ↑ (13.8–30.7 times)	No	No
*8*	N	No	2.5↑	No	No	No	No	No	No	N	Mild ↑ (13.8–30.7 times)	No	No
*9*	42↑	117↑	13.7↑	No	6.5	9.66	16.67	3.93	No	1359↑	↑	↑	↑
*10*	76↑	105↑	4.77↑	No	N	No	No	No	3.894↑	No	6.74↑	No	No
*11*	68↑	57↑	>12↑	No	No	12.36	No	3.41	0.06	89	13.1↑	12.9↑	1.2↑
*12*	49↑	65↑	2.10↑	127↑	5.9	No	No	No	↑	206↑	N	N	N
*13*	84↑	149↑	4.59↑	227.4↑	4.8	75	No						

Note: ALT: alanine aminotransferase; AST: aspartate aminotransferase; C0: free carnitine; C2: acetylcarnitine; C3: propionylcarnitine; C4-DC: succinoylcarnitine; MMA: methylmalonic acid; N: normal; ↑ for elevation
